# Relationship between the degree of recanalization and functional outcome in acute ischemic stroke is mediated by penumbra salvage volume

**DOI:** 10.1007/s00415-021-10410-2

**Published:** 2021-01-24

**Authors:** Gabriel Broocks, Hashim Jafarov, Rosalie McDonough, Friederike Austein, Lukas Meyer, Matthias Bechstein, Noel van Horn, Marie Teresa Nawka, Gerhard Schön, Jens Fiehler, Helge Kniep, Uta Hanning

**Affiliations:** 1 Department of Diagnostic and Interventional Neuroradiology, University Medical Center Hamburg-Eppendorf, Martinistrasse 52, 20246 Hamburg, Germany; 2 Institute of Medical Biometry and Epidemiology, University Medical Center Hamburg-Eppendorf, Hamburg, Germany

**Keywords:** Stroke, Imaging, Computed Tomography, Thrombolysis, Thrombectomy, Ischemia, Infarction

## Abstract

**Background:**

The presence of metabolically viable brain tissue that may be salvageable with rapid cerebral blood flow restoration is the fundament rationale for reperfusion therapy in patients with large vessel occlusion stroke. The effect of endovascular treatment (EVT) on functional outcome largely depends on the degree of recanalization. However, the relationship of recanalization degree and penumbra salvage has not yet been investigated. We hypothesized that penumbra salvage volume mediates the effect of thrombectomy on functional outcome.

**Methods:**

99 acute anterior circulation stroke patients who received multimodal CT and underwent thrombectomy with resulting partial to complete reperfusion (modified thrombolysis in cerebral infarction scale (mTICI) ≥ 2a) were retrospectively analyzed. Penumbra volume was quantified on CT perfusion and penumbra salvage volume (PSV) was calculated as difference of penumbra and net infarct growth from admission to follow-up imaging.

**Results:**

In patients with complete reperfusion (mTICI ≥ 2c), the median PSV was significantly higher than the median PSV in patients with partial or incomplete (mTICI 2a–2b) reperfusion (median 224 mL, IQR: 168–303 versus 158 mL, IQR: 129–225; *p* < 0.01). A higher degree of recanalization was associated with increased PSV (+ 63 mL per grade, 95% CI: 17–110; *p* < 0.01). Higher PSV was also associated with improved functional outcome (OR/mRS shift: 0.89; 95% CI: 0.85–0.95, *p* < 0.0001).

**Conclusions:**

PSV may be an important mediator between functional outcome and recanalization degree in EVT patients and could serve as a more accurate instrument to compare treatment effects than infarct volumes.

**Supplementary Information:**

The online version contains supplementary material available at 10.1007/s00415-021-10410-2.

## Introduction

Mechanical thrombectomy (MT) in acute ischemic stroke substantially improves functional outcome in patients with large vessel occlusion [18, 34]. Yet, the time-sensitive selection of patients who will most likely benefit from MT is a critical factor in clinical practice. Neuroimaging may be used to guide endovascular treatment, and may serve as a prognostic biomarker [1, 2, 35]. Past MT landmark trials including patients 0–6 h from symptom onset applied different brain imaging criteria for treatment selection, for instance using computed tomography (CT) perfusion to estimate ischemic core volume (i.e. volume that is thought to represent irreversible tissue injury), compared to the total volume of hypoperfused brain tissue [1, 22, 28]. Accordingly, the presence of ischemic penumbra (metabolically viable brain tissue that may be salvageable with rapid cerebral blood flow restoration) is the fundamental rationale for reperfusion therapy [11]. However, the effect of endovascular treatment on functional outcome highly depends on the degree of recanalization as exemplified in previous studies [15, 20, 21]. Recently, a meta-analysis found an incremental association between the degree of recanalization and clinical outcome [21]. Currently, the American Heart Association (AHA) guidelines recommend a score of ≥ 2b on the modified Thrombolysis in Cerebral Infarction (mTICI) scale as the angiographic goal of MT [29, 30]. However, a wide range of outcome is still evident even in cases of successful reperfusion, indicating that outcome is completely mediated by further baseline and procedural covariates [9, 21].

Currently, it remains uncertain how the volume of penumbra salvage (PSV) mediates the effect of thrombectomy on functional outcome. Moreover, the relationship of penumbra salvage and the degree of recanalization has not yet been investigated.

We hypothesized twofold: First, a higher degree of recanalization is incrementally associated with higher PSV. Second, we hypothesized that PSV is directly linked to functional outcome.

## Materials and methods

### Patients

For this retrospective study, we consecutively analyzed all ischemic stroke patients with acute large vessel occlusion of the middle cerebral artery admitted between June 2015 and October 2019 at our university hospital, which is a high-volume tertiary stroke center (> 300 stroke thrombectomy procedures per year). Only anonymized data were analyzed after ethical review board approval, and the local ethics committee (Ethikkommission der Ärztekammer Hamburg) waived informed consent after review. The data that support the findings of this study are available from the corresponding author upon reasonable request. The study was conducted in accordance with the ethical guidelines (“Leitlinien der Ärztekammer Hamburg”) of the local ethics committee and in accordance with the Declaration of Helsinki.

All ischemic stroke patients admitted in the aforementioned time period were screened based on the following a priori defined inclusion criteria: (1) acute anterior circulation stroke in the territory of the middle cerebral artery (MCA) and MCA occlusion; (2) multimodal CT imaging protocol at admission including CT Angiography (CTA) and CT Perfusion (CTP); (3) known time window from symptom onset to admission imaging; (4) follow-up CT (FCT) 24 h after admission (max. range 23–25 h after onset); (5) admission National Institutes of Health Stroke Scale (NIHSS) score above 3; (6) documented functional outcome after 3 months based on modified Ranking Scale (mRS) score; (7) Absence of intracranial hemorrhage with significant mass effect (parenchymal hemorrhage (PH) type 2) according to Fiorelli et al. [14] and preexisting thromboembolic or hemodynamic infarctions in admission non-enhanced CT (NECT) or preexisting significant carotid stenosis; (8) Absence of significant motion artifacts.

Only patients fulfilling all criteria were included in this study. Baseline patient characteristics were retrieved from the medical records, including the modified Rankin Scale (mRS) score after 90 days.

Recanalization rates were classified as complete, incomplete and partial recanalization by the responsible neuroradiologists according to the mTICI score; reperfusion grade 2a indicates antegrade reperfusion of less than half of the occluded target artery previously ischemic territory; grade 2b, antegrade reperfusion of more than half of the previously occluded target artery ischemic territory; grade 2c, near-complete perfusion except for slow flow in a few distal cortical vessels or presence of small distal cortical emboli and grade 3, complete antegrade reperfusion of the previously occluded target artery ischemic territory, with absence of visualized occlusion in all distal branches.

Complete recanalization was defined as mTICI 2c/3, based on recent studies recommending this recanalization degree as primary aim of MT [12]. Patients with complete recanalization were compared to patients with successful but incomplete (mTICI 2b), and partial recanalization (mTICI 2a). For dichotomized analysis, patients with complete recanalization were compared to patients with mTICI 2a–2b.

A binary clinical outcome was defined based on modified Rankin Scale (mRS) after 90 days with 0–2 as functional independence and mRS ≥ 3 as poor outcome.

### Image acquisitions

All patients received multimodal stroke imaging at admission with NECT, CTA, and CTP performed in equal order on 256 dual slice scanners (Philips iCT 256). NECT: 120 kV, 280–340 mA, 5.0 mm slice reconstruction, 1 mm increment; CTA: 100–120 kV, 260–300 mAs, 5.0-mm slice reconstruction, 1-mm increment, 80 mL highly iodinated contrast medium and 50 mL NaCl flush at 4 mL/s; CTP: 80 kV, 200–250 mA, 5 mm slice reconstruction (max. 10 mm), slice sampling rate 1.50 s (min. 1.33 s), scan time 45 s (max. 60 s), biphasic injection with 30 ml (max. 40 ml) of highly iodinated contrast medium with 350 mg iodine/ml (max. 400 mg/ml) injected with 6 ml/s, followed by a 30 ml sodium chloride chaser bolus.

### CT-perfusion analysis

**Fig. 1:**
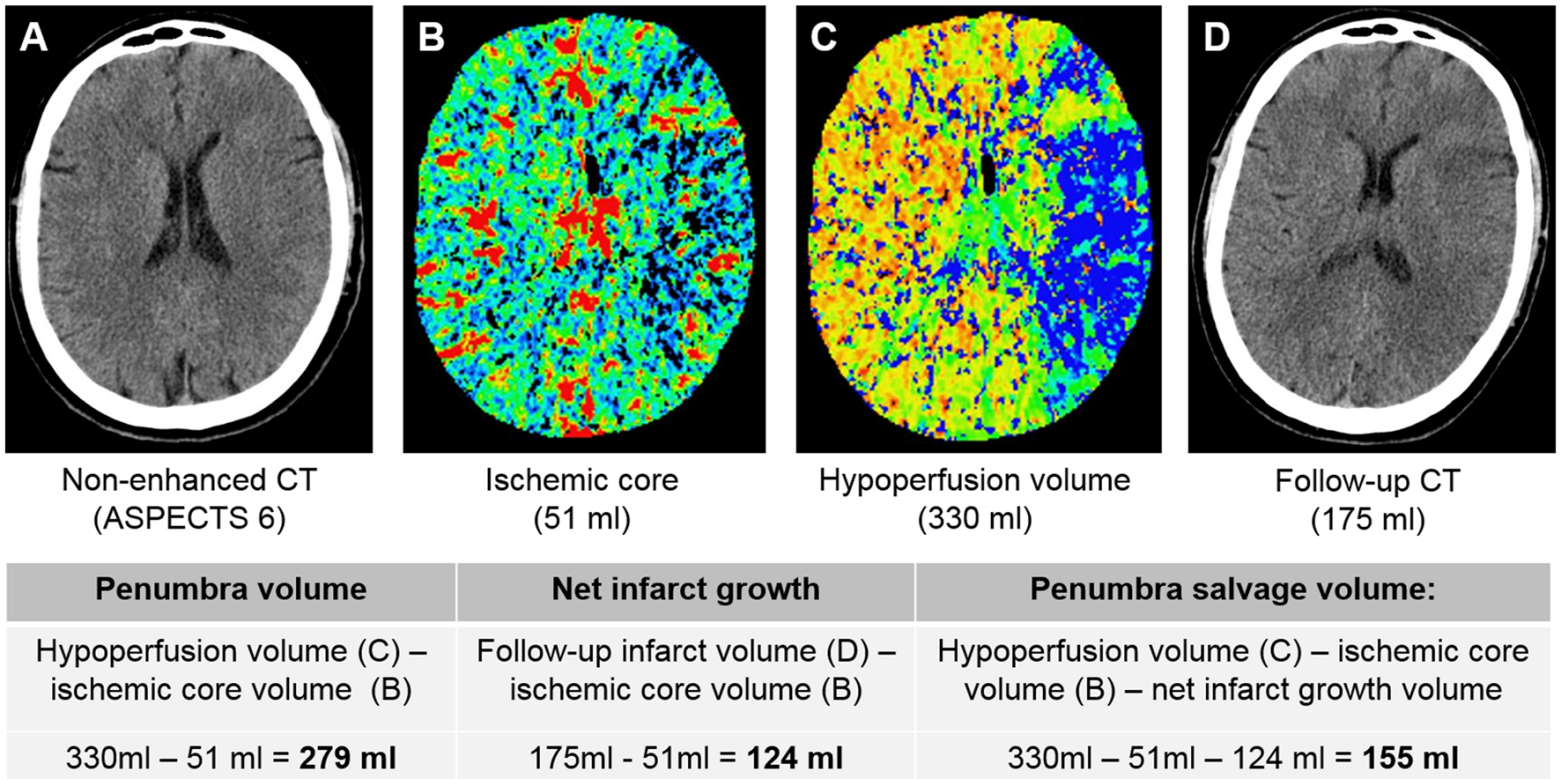
Quantification of penumbra salvage volume. Illustration of the quantification of penumbra salvage volume (PSV). Baseline non-enhanced CT is displayed on the left hand side (**a**), and perfusion imaging besides (**b** for ischemic core, **c** for hypoperfusion volume). Follow-up CT is displayed on the right hand side, where follow-up infarct volume was calculated

Infarct core and penumbra have been assessed using CT-perfusion (CTP) with whole brain coverage. Penumbra has been determined using relative mean transit time (MTT) with a threshold of 145% and infarct core has been defined using absolute cerebral blood volume (CBV) with a threshold at 2.0 ml × 100 g^−1^, as described by Wintermark et al. [36]. Based on the CTP-derived volumes for ischemic core and hypoperfusion volumes, we calculated penumbra volumes as their difference (Eq. 1). Secondly, we determined net infarct growth from admission CT to FCT based on the difference of the total infarct volume in FCT and ischemic core in admission CT (Eq. 2). Finally, we subtracted the net infarct growth volume from penumbra volume to determine penumbra salvage volume (PSV) (Eq. 3). Figure 1 illustrates a case, and how PSV was determined.

1 $$Penumbra volume=V\left(hypoperfusion\right)- V(core)$$

2 $$Net infarct growth=V\left(follow up infarct\right)- V(core)$$

3 $$PSV=V\left(penumbra\right)- V(net infarct growth)$$

Anonymized data was processed at an imaging core lab. Image analysis including volumetric analysis was performed using commercially available software (Analyze 11.0, Biomedical Imaging Resource, Mayo Clinic, Rochester, MN). All analyses were conducted by an experienced neuroradiologist (> 10 years of experience). Subsequently, all cases were screened in a consensus reading with a second experienced neuroradiologist.

### Statistical analysis

**Fig. 2:**
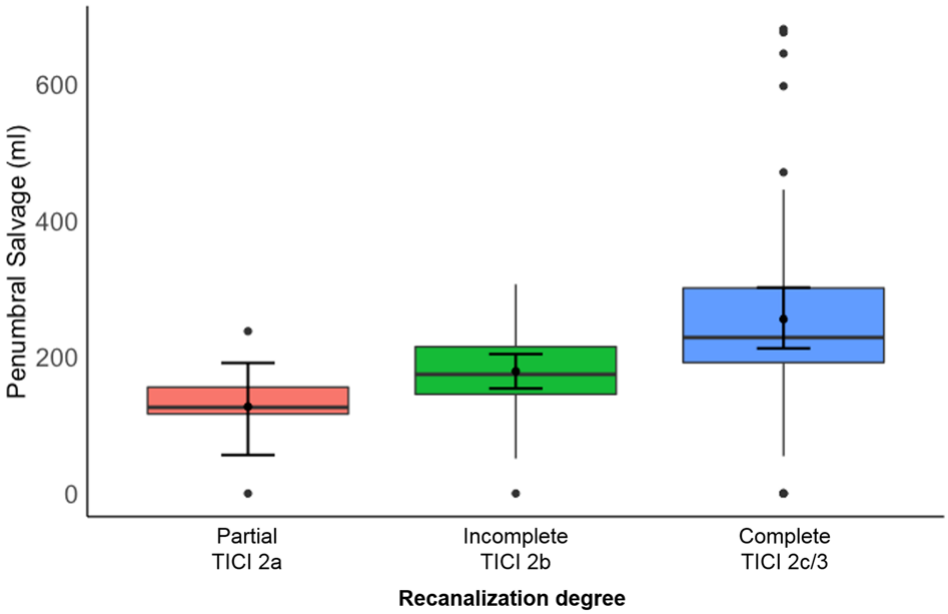
Relationship of recanalization degree and penumbra salvage volume. Illustration of the relationship of recanalization degree, and penumbra salvage volume (y-axis), separately for patients with partial, incomplete, or complete recanalization

Univariable distribution of metric variables is described by median and interquartile range (IQR). Absolute and relative frequencies are given for categorical data. To compare two independent samples regarding a metric or categorical outcome we used Mann–Whitney *U* test or χ2 test, respectively (Table 1). The impact of recanalization degree on PSV was illustrated in boxplots (Fig. 2).

**Table 1:** Patient characteristics

Patient characteristics	Functional independence (mRS 0–2)	Poor outcome (mRS 3–6)	*p* value
Subjects, *n* (%)	39 (39)	60 (61)	
Baseline parameter			
Age in years, median (IQR)	68 (59–78)	77 (69–82)	< 0.01
Female sex, *n* (%)	18 (47)	34 (57)	0.34
Admission NIHSS, median (IQR)	13 (9–17)	17 (15–20)	< 0.001
ASPECTS, median (IQR)	8 (7–9)	8 (6–9)	0.48
Imaging lesion volumes			
Ischemic core volume (mL), median (IQR)	9 (0–33)	19 (5–55)	0.05
Penumbra volume (mL), median (IQR)	211 (188–268)	214 (165–265)	0.71
Follow-up infarct volume (mL), median (IQR)	12 (3–26)	49 (7–120)	< 0.01
Treatment and outcomes			
Intravenous alteplase, *n* (%)	30 (78)	33 (55)	0.02
Time to recanalization in min, median (IQR)	264 (216–382)	308 (255–404)	0.15
Complete recanalization (TICI ≥ 2c), *n* (%)	28 (71)	31 (52)	0.04
Modified Rankin Scale, median (IQR)	1 (0–1)	5 (4–6)	< 0.001
Parenchymal hemorrhage type 1, *n* (%)	2 (4)	3 (5)	0.67
Penumbra salvage volume, median (IQR)	200 (157–253)	190 (121–224)	0.23

**Fig. 3:**
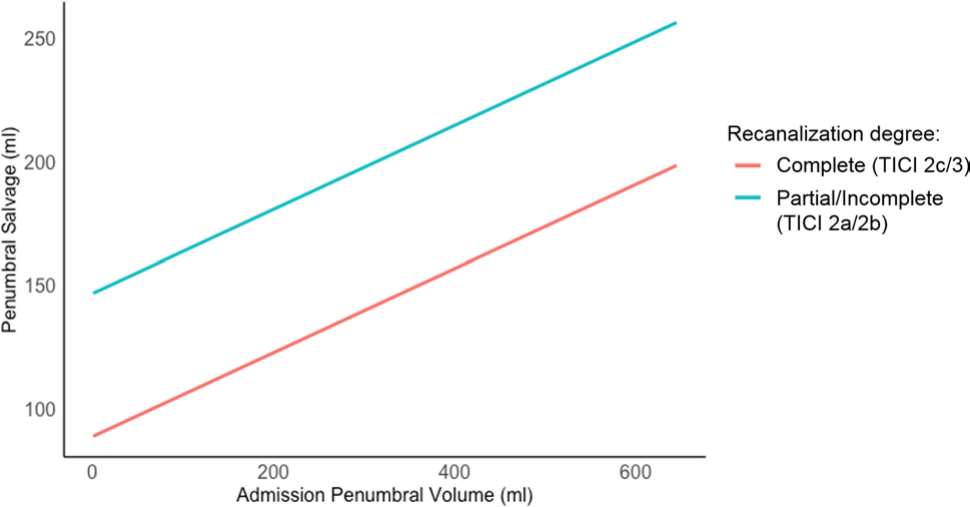
Relationship of baseline penumbra volume, and recanalization degree on penumbra salvage. Impact of a higher recanalization degree on penumbra salvage volume (y-axis), according to the baseline penumbral volume (x-axis)

Univariable and multivariable linear regression analyses were performed with PSV as dependent variable, and age, ASPECTS, core lesion volume, penumbra volume, application of intravenous alteplase, degree of recanalization (partial, incomplete, complete), NIHSS, and time from onset to recanalization as independent parameters. For multivariable analysis, backward selection was used integrating all above-mentioned variables that showed a significant association to PSV in univariable analysis (Table 2). The impact of recanalization degree on PSV according to the baseline penumbra volume is shown in Fig. 3.

**Table 2:** Univariable and multivariable linear regression to predict penumbra salvage volume (PSV) and univariable and multivariable ordinal regression analyses to predict clinical outcome (mRS shift at day 90)

Parameter	*ß*	95% CI	*p* value
Univariable linear regression analysis (Penumbra salvage volume**)**			
Recanalization degree	63.4 mL	16.8–110.1 mL	< 0.01
Hypoperfusion volume (per 1 mL)	0.85 mL	0.72–0.98 mL	< 0.001
Penumbra volume (per 1 mL)	0.88 mL	0.74–1.0 mL	< 0.001
Multivariable linear regression analysis (Penumbra salvage volume)			
Recanalization degree	32.1 mL	8.39–55.8 mL	0.009
Penumbra volume (per 1 mL)	0.83 mL	0.71–1.00 mL	< 0.001
Time onset-recanalization (per minute)	− 0.11 min	− 0.22–0.02 min	0.03

Clinical outcome (mRS shift day 90)			
Parameter	OR	95% CI	*p* value
Univariable ordinal regression analysis (mRS shift day 90)			
Age (per year)	1.04	1.008–1.070	0.012
NIHSS	1.09	1.02–1.16	0.010
ASPECTS	0.71	0.57–0.89	0.003
Ischemic core volume (per 10 mL)	1.11	1.03–1.19	0.005
Complete reperfusion	0.46	0.22–0.97	0.040
Penumbra salvage volume (per 10 mL)	0.96	0.93–0.99	0.019
Multivariable ordinal regression analysis (mRS shift day 90)			
Age	1.07	1.04–1.11	< 0.001
Penumbra salvage volume (per 10 mL)	0.89	0.85–0.95	< 0.0001
Ischemic core volume (per 10 mL)	1.18	1.09–1.28	< 0.001
Penumbra volume (per 10 mL)	1.08	1.02–1.15	0.011
Complete reperfusion	0.41	0.18–0.96	0.039

*NIHSS* National Institute of Health Stroke Scale, *ASPECTS* Alberta Stroke Program Early CT Score, *OR* odds ratio

**Fig. 4:**
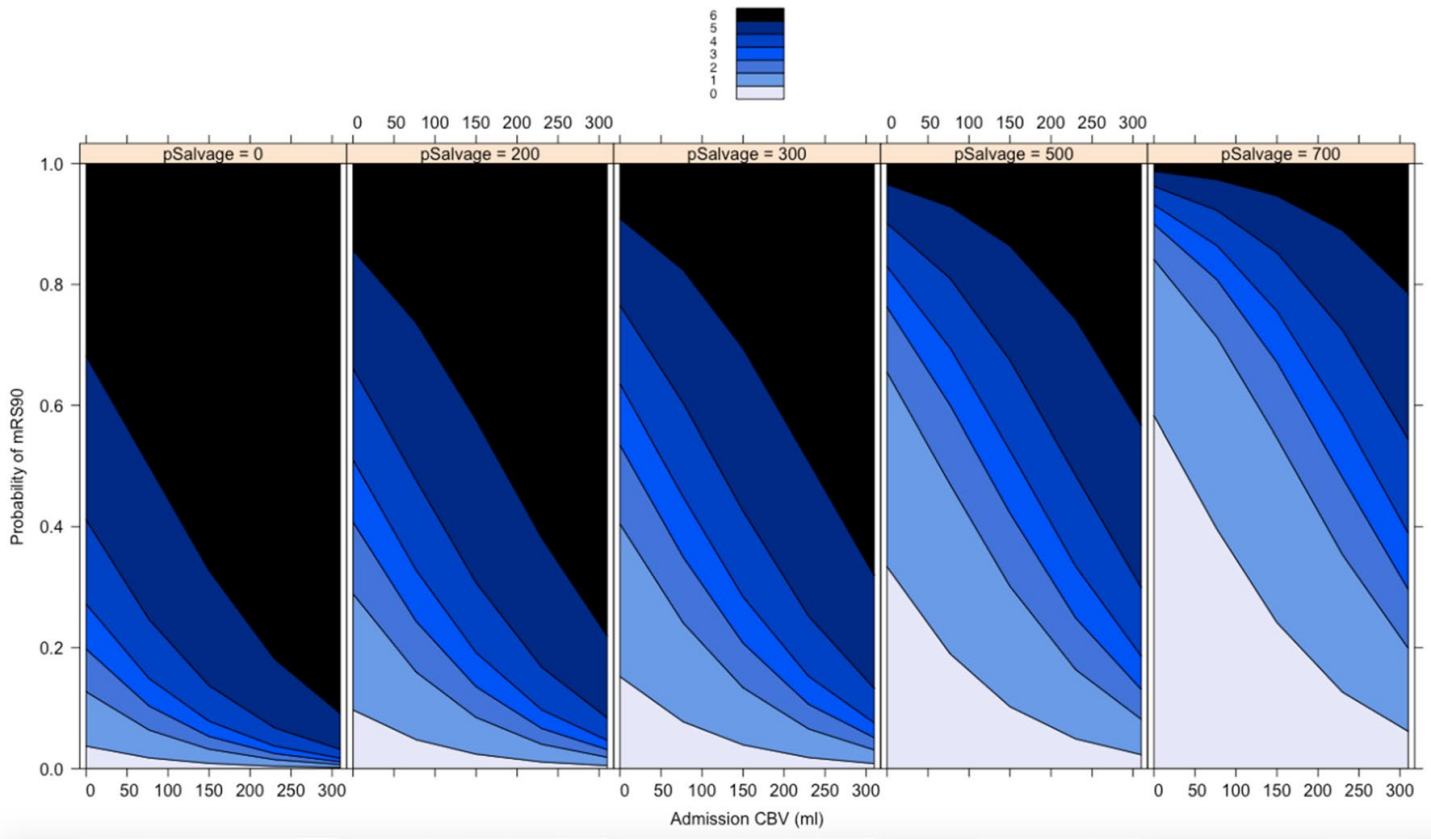
Relationship of the baseline ischemic core volume and penumbra salvage volume, and its impact on functional outcome at 90 days**.** Impact of baseline ischemic core volume (x-axis), and penumbra salvage volume (5 plots from 0 to 700 mL), on probability for mRS at day 90 (0–6, indicated by blue colors)

Secondly, uni- and multivariable ordinal regression analyses were performed with modified Ranking Scale score at day 90 (mRS) as dependent variable using the same aforementioned independent variables. The ordinal form of the day 90 mRS was chosen due to its better relation to long-term outcomes in patients following ischemic stroke than dichotomized mRS [16] (Table 2). Figure 4 shows effect plots for ordinal regression analysis with probability for mRS shift (y-axis) depending on baseline ischemic core volume (x-axis) separately for different levels of PSV. Further effect plots are displayed in the supplemental material (Supplemental Fig. 1 for ASPECTS, Supplemental Fig. 2 for age).

Finally, two multivariate logistic regression models to predict functional independence were tested against each other to determine the additional value of PSV for the prediction of functional outcome. Both models included baseline ischemic core volume, penumbra volume, adjusted for age and degree of recanalization. In model 2, PSV was added as further variable. For both models (model 1 − PSV; model 2 + PSV), the predictive values were plotted against each other using receiver operating characteristic (ROC) curve analyses. Area under curve (AUC) of both models was compared using DeLong test. The dependent variable was functional independence (mRS 0–2 at day 90).

A statistically significant difference was accepted at a *p* value of less than 0.05. Analyses were performed using MedCalc (version 11.5.1.0; Mariakerke, Belgium) and R (R Core Team. R: A Language and Environment for Statistical Computing. R Foundation for Statistical Computing. Vienna, Austria, 2017).

### Data availability statement

The data that support the findings of this study are available from the corresponding author upon reasonable request.

## Results

### Patients

A total of 99 patients fulfilled the inclusion criteria. The median age of the patients was 76 years (IQR: 65–80). 52 (53%) patients were female and 47 (47%) were male. The median NIHSS was 16 (interquartile range (IQR): 12–19) and the initial ASPECTS was 8 (IQR: 7–9). Functional independence at day 90 (mRS 0–2) was observed in 45 patients (45%). Patient characteristics are summarized in Table 1.

All patients underwent MT with partial to complete recanalization, 12 patients with partial (mTICI 2a) (12%), 35 patients with successful but incomplete (mTICI 2b) (35%), and 52 patients (52%) with complete recanalization (16 patients with mTICI 2c, and 36 patients with mTICI3). The median time from onset to recanalization was 291 min (IQR: 233–395 min). At baseline, the median ischemic core volume was 15.6 mL (IQR: 1.2–47.5 mL), and the median penumbra volume was 213.7 mL (IQR: 175.5–265.0 mL).

Comparing patients with functional independence at day 90 (mRS 0–2) to patients with an mRS 3–6, we observed that patients with functional independence were younger (68 versus 77 years) (*p* < 0.01), and showed a lower NIHSS on admission (13–17) (*p* < 0.001). On baseline imaging, ischemic core volume was by trend lower (9–19 mL) (*p* = 0.71) in the patients with functional independence at day 90. Furthermore, patients with functional independence received intravenous alteplase more often (78 versus 55%) (*p* = 0.02), and the degree of recanalization after MT was higher (complete recanalization 71 versus 52%) (*p* = 0.04). Total infarct volume in FCT was lower in patients with functional independence (12–49 mL) (*p* < 0.01) (Table 1).

In patients with complete recanalization, the median PSV was significantly higher than the median PSV in patients with partial or incomplete recanalization (median 224 mL, IQR: 168–303 versus 158 mL, IQR: 129–225; *p* < 0.01). Correspondingly, the median relative penumbra salvage (proportion of rescued penumbra/total penumbral volume at baseline) was 95% (IQR: 77–99%) for patients with complete recanalization, which was significantly higher than the median relative penumbra salvage in patients with partial or incomplete recanalization (82%, IQR: 56–96%; *p* = 0.04).

### Penumbra salvage volume—linear regression analyses

In univariable linear regression analysis, degree of recanalization, hypoperfusion volume, and penumbra volume were significantly associated with PSV as dependent parameter (Table 2). In multivariable linear regression analysis, degree of recanalization (*ß* = 32.1; *p* = 0.009), penumbra volume (per 1 mL) (*ß* = 0.83; *p* < 0.001), and time from symptom onset to recanalization (*ß* = − 0.11, *p* = 0.03), were significantly and independently associated with PSV (Table 2). There was no association between age and PSV, and no association between baseline core lesion volume and PSV.

### Functional outcome—ordinal regression analyses

In univariable ordinal regression analysis, age, ASPECTS, baseline NIHSS, ischemic core volume, PSV, and degree of recanalization were significantly associated with mRS shift at day 90 as dependent parameter (Table 2, lower part). In multivariable ordinal regression analysis, PSV (odds ratio: 0.89; *p* < 0.0001), penumbra volume (odds ratio: 1.08; *p* = 0.011), ischemic core lesion volume (per mL) (odds ratio: 1.18; *p* < 0.001), age (odds ratio: 1.07; *p* < 0.001), and degree of recanalization (odds ratio: 0.41; *p* = 0.039) were significantly and independently associated with mRS shift at day 90 (Table 2, lower part). Time from symptom onset to recanalization, and baseline NIHSS were not significantly associated with outcome.

### Multivariable prediction model

The AUC for model 1 (− PSV) to classify functional independence (mRS 0–2 at day 90) was 0.71 (95% CI: 0.60–0.80; *p* < 0.001). The AUC for model 2 (+ PSV) to classify functional independence was 0.80 (95% CI: 0.70–0.88; *p* < 0.0001). In pairwise comparison using DeLong tests, we observed a significant difference between both models (difference 0.09, 95% CI: 0.02–0.16; *p* = 0.015).

## Discussion

Our study on the relationship of recanalization degree and PSV, and its impact on functional outcome revealed several findings: (1) higher degree of recanalization was directly associated with increased PSV: every increase in reperfusion (partial (mTICI 2a), incomplete (mTICI 2b), and complete (mTICI ≥ 2c) recanalization), was associated with a 63 mL increased PSV; (2) that was associated with improved functional outcome at 90-days follow-up; (3) this effect was shown even when comparing patients with mTICI 2b to patients with mTICI ≥ 2c.

In detail, we observed that penumbra salvage depends on three parameters: penumbra volume at baseline, degree of recanalization, and time from onset to reperfusion. However, penumbra salvage was independent from baseline ischemic core volume and ASPECTS, highlighting that penumbra may be rescued even in patients presenting with extensive stroke at admission. Furthermore, we observed that higher PSV and complete reperfusion were significantly and independently associated with improved functional outcome at 90-days. Corroborating previous studies, baseline ischemic core volume, and age had a significant impact on functional outcome [13]. Nevertheless, a higher degree of penumbra salvage might lead to a better clinical outcome even in older patients, or patients with large baseline ischemic core, as exemplified in Fig. 4 (see also supplemental figures for age effect plot) [11, 23, 24]. Interestingly, time from onset to reperfusion had no significant impact on functional outcome, illustrating that patients in the extended time window may benefit from endovascular treatment [1]. Although the impact of higher recanalization degree on functional outcome is well-established, penumbra salvage may improve outcome prediction, as exemplified by comparing two multivariable predictive models. A model that included PSV exhibited a significantly better diagnostic ability to classify functional outcome (AUC: 0.71 versus 0.80).

Penumbra salvage may be considered as a measure of success of MT, hence associated with functional outcome. Yet, the effect of endovascular treatment on clinical outcome is not completely understood. Contributing factors beyond reperfusion, including the underlying pathophysiology such as magnitude of immanent tissue injury, collateral circulation, clinical variables, or subsequent developments like recurring stroke or secondary hemorrhage, reasonably influence the clinical outcomes [11, 31]. Additionally, the effect of MT on outcome may not only be attributed to penumbral salvage, but also on reducing secondary injury volumes, in particular ischemic brain edema [8–10, 19, 32]. To illustrate this, we observed that complete reperfusion was associated with a penumbra salvage of 74 mL. Estimating the effect of PSV on mRS at day 90 in linear regression, a PSV of 74 mL would equal a decrease in mRS of 0.22. Complete reperfusion, however, was associated with a lower mRS of 0.95. Therefore, the effect of successful reperfusion on clinical outcomes is not comprehensively explained by penumbral salvage and may be multifactorial. Lately, it has been observed that successful MT was associated with a reduced ischemic formation of 6.3%, and improved mRS at day 90 of − 1.1 [8]. Thus, edema reduction may be an explanation of the discrepancy between outcome improvement and penumbra salvage following MT [8, 9, 26].

So far, it is well known that increasing reperfusion is directly associated with improved functional outcome [20, 21]. A recent analysis of the HERMES data observed the increasing rate of favorable outcome with increasing degree of recanalization [21]. A further recent study observed that even in the subgroup of patients with “successful” MT (i.e. mTICI ≥ 2b) the highest possible reperfusion grade should be pursued [20]. However, both studies did not discuss any pathophysiological reasons regarding the relationship of functional outcome and reperfusion degree.

To our knowledge, this is the first study that directly quantified the volume of penumbra salvage and investigated its relationship to the degree of recanalization and functional outcome. This study might help to better understand how endovascular treatment effects outcome, and how to further improve functional outcome in patients. Additionally, PSV could be tested as an imaging biomarker to compare treatment effects in ischemic stroke, as measuring infarct volume in follow-up imaging may not be an optimal parameter for this concern [5]. A previous study observed, that reduced infarct volume in follow-up imaging after MT only explained 12% of the treatment benefit [5]. However, this study did not describe baseline ischemic core volume, or penumbral volume, which might represent a major limitation of that study.

Future studies may investigate whether penumbra salvage is a better mediator of the relationship of endovascular treatment and functional outcome. Furthermore, it is important to realize that in the referred study, infarct volume was derived in follow-up imaging that has been acquired between 12 h to 2 weeks after admission. This directly impairs the interindividual comparability of lesion volumes due to the significantly ranging proportion of ischemic edema. At 24 h after onset, it has been observed that edema contributes to approximately 30% of the total lesion, while after 12 h, the mean edema proportion is around 20% [7, 17, 33]. This proportion, however, significantly varies depending on time, reperfusion treatment, and individual progression [7, 25, 27]. Consequently, future research is needed to investigate how edema-corrected lesion volumes perform as a mediator between outcome and EVT, and how these volumes may improve the comparability of treatment effects.

## Limitations

Limitations of this study include the relatively small number of patients, due to rigorous inclusion criteria. The intention was to obtain a homogenous patient cohort. Patients with parenchymal hemorrhage type 2 were excluded. Future studies could investigate the relationship of PSV and secondary hemorrhage. Furthermore, there is no coherent definition of the true ischemic core and penumbra, and this concept has its natural limitations [6]. Alternative approaches could use relative cerebral blood flow to define ischemic core, but this may indicate a higher occurrence of core volume overestimation [3, 4].

## Conclusion

Penumbra salvage volumes increased with higher degrees of recanalization and were significantly associated with improved functional outcome at day 90. These results further emphasize the importance of complete reperfusion as a result of EVT. Penumbra salvage was independent from baseline ischemic core volume, highlighting that penumbra may be rescued even in patients presenting with extensive ischemic core at admission.

## Supplementary Information

Below is the link to the electronic supplementary material.

Supplementary file1 (PDF 437 KB)
