# Supplementary material for: Relationship between the degree of recanalization and functional outcome in acute ischemic stroke is mediated by penumbra salvage volume
Source: J Neurol. 2021 Jan 24;268(6):2213–22. doi: 10.1007/s00415-021-10410-2 (PMC8179901; doi:10.1007/s00415-021-10410-2)

SUPPLEMENTAL MATERIAL

Supplemental Figure 1 - Relationship of the baseline ischemic core volume indicated by ASPECTS and penumbra salvage volume, and its impact on functional outcome at 90 days.

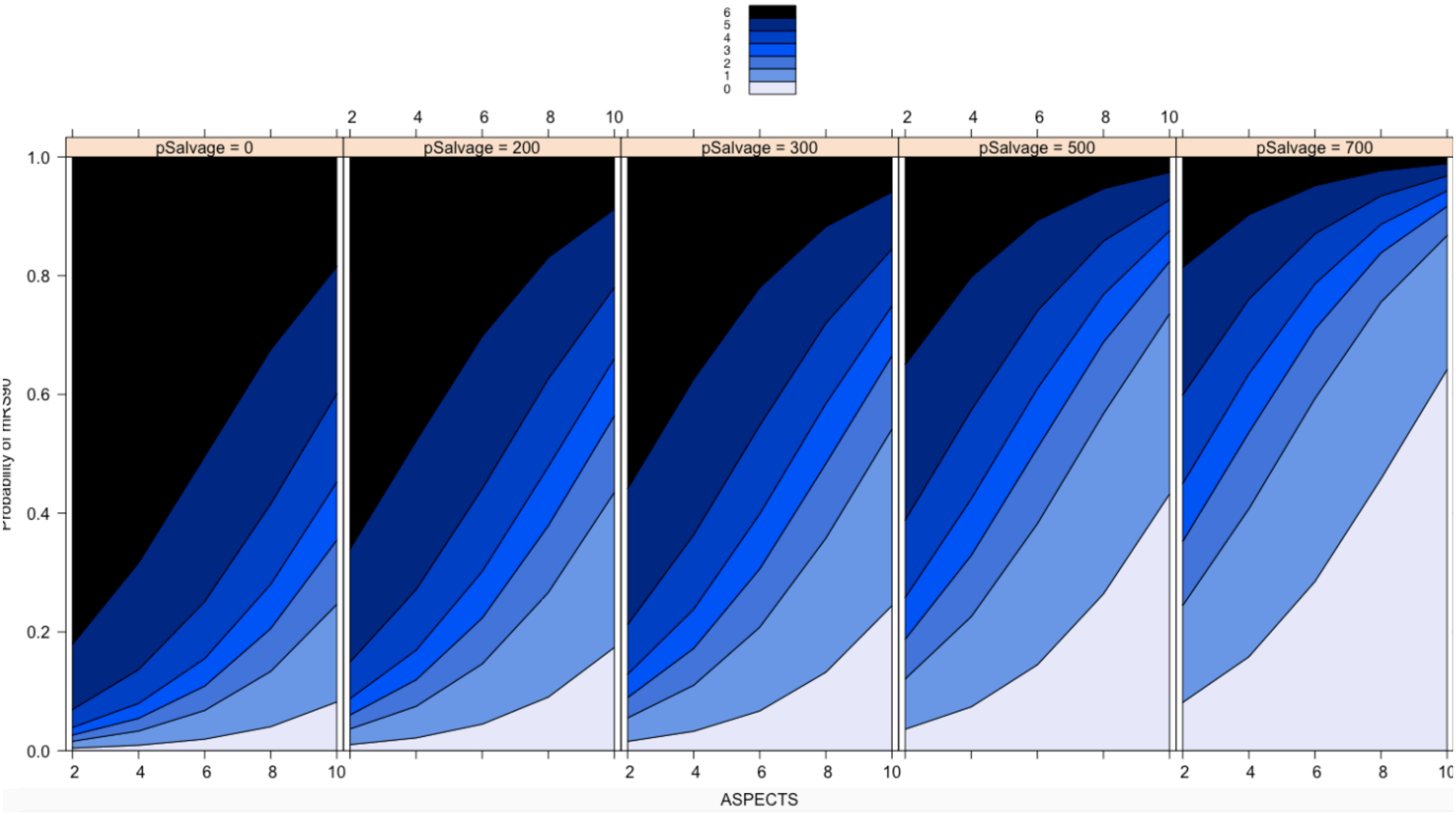

Supplemental Figure 2 - Relationship of age and penumbra salvage volume, and its impact on functional outcome at 90 days.

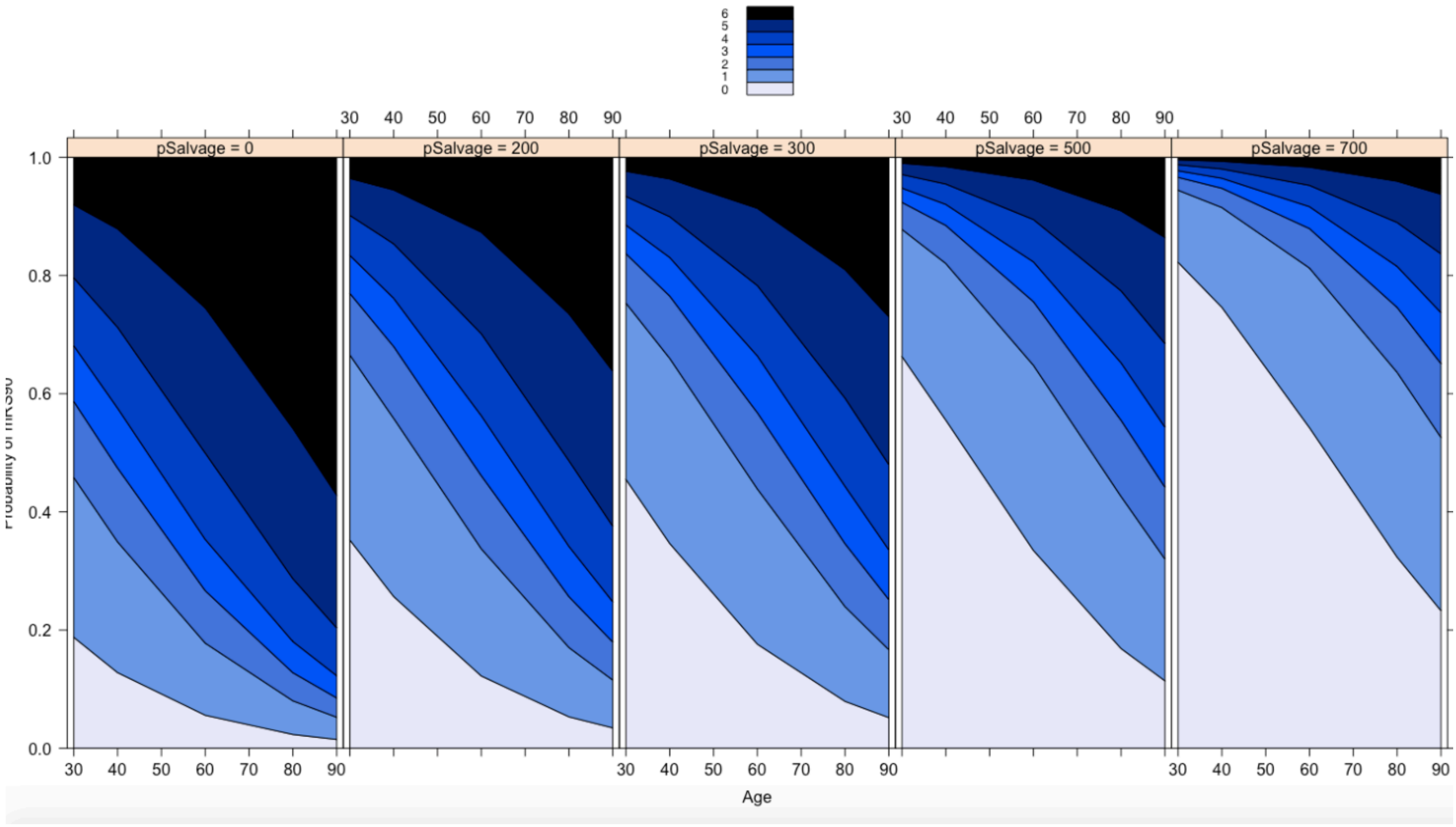

Supplement: Supplementary file 1 — Supplementary file1 (PDF 437 KB) [file 415_2021_10410_MOESM1_ESM.pdf]
